# Noninvasive prenatal testing complicated by maternal malignancy: new tools for a complex problem

**DOI:** 10.1038/npjgenmed.2015.2

**Published:** 2016-01-13

**Authors:** Y M Dennis Lo

**Affiliations:** 1 Li Ka Shing Institute of Health Sciences and Department of Chemical Pathology, The Chinese University of Hong Kong, Prince of Wales Hospital, Hong Kong SAR, China

The discovery of cell-free foetal DNA in maternal plasma in 1997 has opened up new possibilities for noninvasive prenatal testing (NIPT).^1^ Since 2011, this technology has been used by over two million pregnant women across the world for the NIPT of common trisomies.^2^ Regions with a particular high rate of uptake include the USA, China and parts of Europe. With the increased use of NIPT, there have been a recent flurry of reports which suggest that such testing, in addition to informing one of the genetic well-being of the foetus, would also occasionally give information regarding the health of the pregnant mother.^3^ In particular, there have been a number of publications reporting that NIPT would reveal the presence of maternal malignancies.^4–6^ In particular, Osborne *et al.* reported a pregnant woman with concurrent metastatic malignancy. NIPT results from this pregnant women on two occasions revealed data suggestive of foetal trisomy 13 and monosomy 18. However, following amniocentesis, the foetal karyotype was found to be normal. Subsequent clinical course and investigation revealed that the woman had metastatic small cell carcinoma, probably of vaginal origin. On retrospect, it was perhaps not surprising that NIPT would reveal maternal malignancy as, prior to the report by Osborne *et al.*, it had been reported by a number of groups that genomewide massively parallel sequencing of plasma DNA could revealed cancer-associated copy-number aberrations in a number of cancer types.^7–9^ It has been estimated that malignancy occurs in ~1 in 1,000 pregnant women.^10^ Hence, one would expect that it would be a matter of time before a pregnant subject opting for NIPT would also be found to have concurrent malignancy.

Amant *et al.* recently reported their experience with applying NIPT to over 4,000 pregnant women.^4^ They identified three pregnant women who had aberrant genomic profiles in their plasma, which could not be traced back to the corresponding fetuses. Through the use of whole-body magnetic resonance imaging, an ovarian carcinoma (International Federation of Gynecology and Obstetrics stage IV-A), a follicular lymphoma (Ann Arbor stage II-SE) and a Hodgkin lymphoma (Ann Arbor stage II) had been found. Bianchi *et al.* reported a larger cohort of 125,426 pregnant women who had undergone NIPT.^5^ Three thousand seven hundred and fifty-seven subjects had aberrant plasma DNA profiles. Amongst these, the referring clinician subsequently and voluntarily reported 10 cases of maternal cancer, including 3 cases of B-cell lymphoma (one stage II, one stage IV and one stage IVB), 1 case each of T-cell leukaemia (stage unknown), Hodgkin lymphoma (stage IIA), unspecified adenocarcinoma (stage not reported), leimyosarcoma (stage not reported), neuroendocrine carcinoma (stage IV, metastatic), colorectal carcinoma (stage IIIC) and anal carcinoma (stage IIIB). Bianchi *et al.* performed additional sequencing and bioinformatic analyses on 8 cases. They found that 7 of the 8 cases had plasma DNA aberrations involving more than one chromosome.

Taken as a whole, NIPT is capable of uncovering previously unknown cases of malignancies in pregnant women. It appears that most of the maternal cancer cases were relatively late-stage disease, i.e., stages III and IV. This information should be disseminated to clinicians and genetic counsellors involved in NIPT and should be included in consent forms for NIPT. The sensitivity of NIPT for identifying maternal cancer, however, requires more exploration. For example, the data from Amant *et al.* indicate that they were able to detect maternal cancers in ~0.1% of their studied subjects. This percentage is similar to the figure concerning the incidence of malignancy in pregnant subjects.^10^ On the other hand, the data from Bianchi *et al.* show that in their cohort, maternal cancer cases were only reported in 0.008% of cases, which is 12.5 times lower than the data from Amant *et al.* However, it is important to note that the designs of the studies by Amant *et al.* and Bianchi *et al.* are different. For example, Amant *et al.* followed up the cases with aberrant plasma DNA profiles not attributable to the fetuses using whole-body diffusion-weighted magnetic resonance imaging. On the other hand, Bianchi *et al.* relied on the referring clinicians to report cases of maternal cancer. It is thus possible that cases of maternal cancer might not present or be identified within the study timeframe. Furthermore, it is also important to bear in mind that differences in the sequencing and bioinformatic protocols of the these studies might also contribute partly to the observed results.

In terms of technological developments, it would be ideal if future generations of NIPT would allow one to identify the tissue of origin of genomic aberrations observed in plasma. In this regard, Sun *et al.* have recently developed such a technology.^11^ This approach takes advantage of the fact that different tissues in the body contain different DNA methylation patterns.^12^ Hence, following genomewide bisulfite sequencing of plasma DNA,^13,14^ one could analyse the data with the help of a set of over 5,800 DNA methylation markers which provide tissue-related methylation information. Using a process of deconvolution, one can then deduce the proportional contributions of the most important tissue types that are releasing DNA into plasma. This approach has been referred to as plasma DNA tissue mapping.^11^ Sun *et al.* demonstrated that the white blood cells, liver and placenta are major tissue contributors to the circulating DNA pool in pregnant women.

Sun *et al.* further reasoned that for a tumour that is releasing its DNA into the plasma, genomic regions in which the tumour cells have copy-number gains (i.e., amplifications) would result in an increase in the proportional representation of the tissue type of the cancer in plasma. Conversely, for genomic regions in which the tumour cells have copy-number losses (i.e., deletions), the proportional representation of the tissue type of the cancer in plasma would decrease. Hence, by systematically comparing the tissue-related contributions of regions showing amplifications and deletions in plasma for different tissue types, one could identify the likely sources of such plasma DNA aberrations. Sun *et al.* illustrated this principle by applying this technology to a pregnant woman undergoing NIPT who exhibited plasma DNA copy-number aberrations in multiple chromosomes.^11^ Using plasma DNA tissue mapping, Sun *et al.* found an unusually highly contribution from B-cells into the plasma DNA pool and determined that B-cells were the likely source of the observed copy-number aberrations in plasma. These data were consistent with the clinical and histological diagnosis of follicular lymphoma, in which the tumour cells were of B-cell origin. These data would need to be validated in a larger cohort.

Through the use of plasma DNA tissue mapping in different clinical scenarios, one could obtain an idea of the spectrum and potential changes in the major tissue contributors to the plasma DNA pool in different clinical settings. Outside of the NIPT context, such an approach would be useful for investigators interested in using plasma DNA analysis for noninvasive cancer detection and monitoring. Currently, plasma DNA tissue mapping is still relatively expensive and involved genomewide bisulfite sequencing. The use of a more targeted approach would be expected to reduce the cost of the process, thus enhancing its potential clinical application. Plasma DNA tissue mapping can be regarded as a technology that links information generated using liquid biopsies with anatomical location.
